# Presence of periaortic gas in *Clostridium septicum*-infected aortic aneurysm aids in early diagnosis: a case report and systematic review of the literature

**DOI:** 10.1186/s13256-017-1422-0

**Published:** 2017-09-21

**Authors:** Fumihito Ito, Ryota Inokuchi, Akinori Matsumoto, Yoshibumi Kumada, Hideyuki Yokoyama, Tokiya Ishida, Katsuhiko Hashimoto, Masashi Narita, Kazuaki Shinohara

**Affiliations:** 1 Department of Emergency and Critical Care Medicine, Ohta Nishinouchi Hospital, 2-5-20 Nishinouchi, Koriyama, Fukushima 963-8558 Japan; 2 Department of Emergency and Critical Care Medicine, JR General Hospital, 2-1-3 Yoyogi, Shibuya-ku, Tokyo, 151-8528 Japan; 3 Department of Emergency and Critical Care Medicine, The University of Tokyo Hospital, 7-3-1 Hongo, Bunkyo-ku, Tokyo, 113-8655 Japan; 4 Department of Infectious Diseases, Okinawa Chubu Hospital, 281 Miyazato, Uruma, Okinawa 904-2293 Japan

**Keywords:** Infected aortic aneurysm, Aortic rupture, Aortic dissection, Sepsis, Septic shock, *Clostridium* spp, Colon adenocarcinoma, Adult

## Abstract

**Background:**

*Clostridium septicum*-infected aortic aneurysm is a fatal and rare disease. We present a fatal case of *C. septicum*-infected aortic aneurysm and a pertinent literature review with treatment suggestions for reducing mortality rates.

**Case presentation:**

A 58-year-old Japanese man with an unremarkable medical history presented with a 3-day history of mild weakness in both legs, and experienced paraplegia and paresthesia a day before admission. Upon recognition of signs of an abdominal aortic aneurysm and paraplegia, we suspected an occluded Adamkiewicz artery and performed a contrast-enhanced computed tomography scan, which revealed an aortic aneurysm with periaortic gas extending from his chest to his abdomen and both kidneys. Antibiotics were initiated followed by emergency surgery for source control of the infection. However, owing to his poor condition and septic shock, aortic repair was not possible. We performed bilateral nephrectomy as a possible source control, after which we initiated mechanical ventilation, continuous hemodialysis, and hemoperfusion. A culture of the samples taken from the infected region and four consecutive blood cultures yielded *C. septicum.* His condition gradually improved postoperatively; however, on postoperative day 10, massive hemorrhage due to aortic rupture resulted in his death.

**Conclusions:**

In this patient, *C. septicum* was thought to have entered his blood through a gastrointestinal tumor, infected the aorta, and spread to his kidneys. However, we were uncertain whether there was an associated malignancy.

A literature review of *C. septicum*-related aneurysms revealed the following: 6-month mortality, 79.5%; periaortic gas present in 92.6% of cases; no standard operative procedure and no guidelines for antimicrobial administration established; and *C. septicum* was associated with cancer in 82.5% of cases.

Thus, we advocate for early diagnosis via the identification of periaortic gas, as an aortic aneurysm progresses rapidly. To reduce the risk of reinfection as well as infection of other sites, there is the need for concurrent surgical management of the aneurysm and any associated malignancy. We recommend debridement of the infectious focus and *in situ* vascular graft with omental coverage. Postoperatively, orally administered antibiotics must be continued indefinitely (chronic suppression therapy).

We believe that these treatments will decrease mortality due to *C. septicum*-infected aortic aneurysms.

## Background

*Clostridium septicum* is an anaerobic, spore-forming, toxin-producing Gram-positive bacillus with peritrichous flagella that only accounts for 1.3% of all clostridial infections. However, the sepsis caused by this organism is severe and has an overall mortality rate of approximately 60% [1]. The clinical relevance of this form of sepsis lies in its association with gastrointestinal malignancy, with the most common being adenocarcinoma of the right colon [2–4]. It is thought that this organism enters the blood through gastrointestinal tumors and infects the aorta.

Although *C. septicum* is a known cause of gas gangrene, it is a very rare cause of an aortic aneurysm. Why this organism favors aortic infection and how clostridial mycotic aneurysms form are not fully understood. It is thought that ulcerative lesions of the gastrointestinal tract, especially colon carcinoma, allow the organism to enter the bloodstream, followed by seeding of an atheromatous focus in the aorta [5]. Seeding of the aorta by this organism can often lead to the rapid development of an infected aneurysm, which is uniformly fatal in the absence of surgical intervention [6, 7]. Here we present a fatal case of *C. septicum*-infected aortic aneurysm. To enable the early diagnosis and identification of effective treatment options, we review cases of *C. septicum*-infected aortic aneurysm from the literature.

## Case presentation

**Fig. 1:**
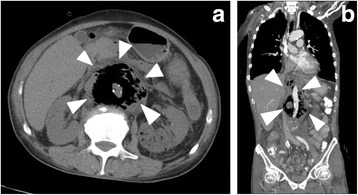
**a** Axial view, and **b** coronal view contrast-enhanced computed tomography scan images showing an aneurysm with periaortic gas (the area immediately outside the gas-filled region is the tunica adventitia of the aorta; shown by *arrowheads*)

A 58-year-old Japanese man complained of mild weakness in both legs 3 days prior to hospitalization. He was admitted to our hospital because he was unable to move his legs and had lost sensation in both legs 1 day prior to hospitalization. He had no remarkable medical history. Although he was lucid, he had a blood pressure of 99/67 mmHg, pulse of 110 beats per minute (bpm), body temperature of 36.1 °C, respiratory rate of 28 breaths/minute, and paralysis and numbness below the T11 level. Because we recognized signs of an abdominal aortic aneurysm and paraplegia on physical examination, we suspected an occlusion of the artery of Adamkiewicz and so performed a contrast-enhanced computed tomography (CT) scan. It revealed the formation of an aneurysm with gas in the aortic wall extending from his chest to his abdomen as well as in both kidneys (Fig. 1); no malignant tumor was detected. Lumbar magnetic resonance imaging also revealed a spinal cord infarction below the T11 level. We initiated antibiotic therapy with 9 g/day ampicillin/sulbactam, 1800 mg/day clindamycin, and 120 mg/day gentamycin; during this period, metronidazole was not available for intravenous injection in Japan. We then performed an emergency surgery for source control of the infection. However, owing to his poor general condition and septic shock, surgery for the mycotic aneurysm was deemed impossible. Thus, we performed bilateral nephrectomy as a source control, and postponed the surgery for the aortic aneurysm until his condition had stabilized. After the bilateral nephrectomy, we initiated mechanical ventilation, continuous hemodialysis, and hemoperfusion treatment with polymyxin B-immobilized fiber (PMX-DHP; Toray Medical Co., Tokyo, Japan).

On postoperative day 3, we began gradually tapering the doses of dopamine and noradrenaline. On postoperative day 5, we transitioned from continuous hemodialysis to intermittent hemodialysis. A culture of the samples taken from the infected region and four consecutive blood cultures revealed *C. septicum*. On postoperative day 6, we performed extubation, after which his condition gradually improved. However, on postoperative day 7, the onset of respiratory muscle fatigue necessitated reintubation. We resumed dopamine on day 9. We planned to operate on the aortic aneurysm; however, his condition deteriorated rapidly. On postoperative day 10, 1500 mL of blood was lost within approximately 20 minutes from the drain; as a consequence, he died. The cause of the hemorrhage was considered to be an aortic rupture. An autopsy was not performed.

## Discussion

In the present case, *C. septicum* was thought to have entered the blood from a gastrointestinal tumor, infected the aorta, and spread from the aorta to the kidneys. However, we were not certain whether this case was associated with cancer.

We searched the Embase®, MEDLINE®, Web of Science, and Google Scholar databases from their inception until 31 December 2015, for case reports (English language articles only) regarding *C. septicum*-related aneurysms. We used the following search terms: ‘*Clostridium septicum*’ and ‘aneurysm’ or ‘aortitis.’ Two reviewers (F.I. and R.I.) independently screened the study titles and abstracts to identify relevant articles for inclusion. The reference lists of the selected articles were also examined for additional publications suitable for inclusion. A consensus for discrepancies between the two reviewers was reached through a discussion with a third reviewer (K.S.). We excluded cases without evidence of an aneurysm with *C. septicum*, cases without an association with *C. septicum*, and academic conference abstracts. The earliest report found in our search was published in 1981.

**Fig. 2:**
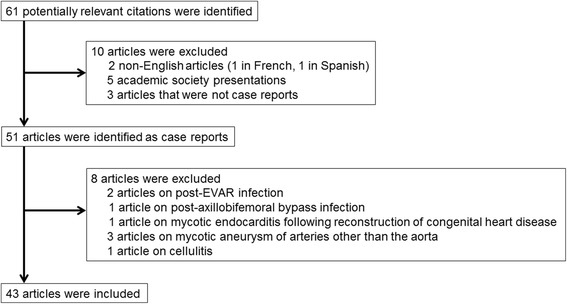
Procedures used for selection of case reports. *EVAR* endovascular aortic repair

The search yielded 61 articles, and 10 articles were excluded: two non-English articles (one in French and one in Spanish), five academic society presentations, and three articles that were not case reports). Of the remaining 51 articles, we excluded two involving post-endovascular aortic repair (EVAR) infection [8, 9], one involving post-axillobifemoral bypass infection [10], one involving mycotic endocarditis following the reconstructive treatment of congenital heart disease [11], three involving the mycotic aneurysm of arteries other than the aorta [12–14], and one involving cellulitis [15], leaving us with 43 articles. In addition to our case, we found a total of 46 cases (Fig. 2).

### Summary of results

A summary of all case reports identified is presented in Table 1 [6, 7, 16–56]. The 6-month mortality rate associated with *C. septicum*-related aneurysms was 79.5%; of the cases, 92.6% showed the presence of periaortic gas, and surgery for a mycotic aneurysm was performed in 52.2% of the cases. The 6-month mortality rate was 66.7% for cases in which elective surgery was not possible and sufficient antimicrobial agents could therefore not be administered prior to surgery. Among the cases in which elective surgery and sufficient preoperative administration of antimicrobial agents were possible, the 6-month mortality rate was 33.3%. In cases in which surgery was not performed, the 6-month mortality rate was 100%.

**Table 1:** Systematic review of *Clostridium septicum*-infected aortic aneurysm cases reported in the English literature

First author and Reference number	Year of publication	Age	Sex	Process	Location	Neoplasm	Surgery	Outcome	Name, duration of antibiotics after surgery for infectious aortic aneurysm
Bridges [16]	1981	68	M	Aneurysm	Infrarenal	Unknown	Axillobifemoral bypass, omental patch	Alive, duration unknown	Penicillin → erythromycin, several months
Semel [17]	1984	60	F	Aneurysm	Ascending/arch	Transverse colon adenocarcinoma	Partial colectomy	Died 20 hours postoperation (cardiac tamponade)	
Momont [18]	1989	85	F	Dissection/aneurysm	Ascending/arch	Cecal adenocarcinoma	None	Died	
Brahan [19]	1990	70	F	Aneurysm	Arch/descending	Ascending colon adenocarcinoma	*In situ* graft, resection of pulmonary artery to aneurysm fistula	Alive at 36 months	Penicillin G 5 weeks → oral penicillin, duration unknown
							Right hemicolectomy		
Hurley [20]	1991	67	M	Aneurysm	Infrarenal	Rectal, colonic adenomas	Right axillobifemoral bypass, aneurysm resection, omental flap	Alive at 9 months	Penicillin G 6 weeks → oral penicillin and clindamycin, duration unknown
							Left due to infected right graft, polypectomy		
Christensen [21]	1993	74	F	Aneurysm	Juxtarenal	None	None	Died	
Messa [22]	1995	77	M	Double aneurysm	Descending thoracic/juxtarenal	Sigmoid adenocarcinoma	*In situ* thoracic graft with omental flap and axillobifemoral bypass	Alive at 4 months	Penicillin, duration unknown → oral penicillin for chronic suppression therapy
Murphy [23]	1996	78	M	Aneurysm	Proximal descending thoracic	Benign sigmoid polyps	*In situ* graft and esophagectomy	Alive at 6 months	Penicillin and clindamycin, duration unknown
Sailors [24]	1996	74	F	Aneurysm	Thoracoabdominal	None	*In situ*	Died 13 weeks postoperatively (pseudoaneurysm rupture)	Vancomycin and ampicillin/sulbactam, 5 days → penicillin G, 12 weeks total
								during a distal anastomosis 14 weeks later	
Monsen [25]	1997	81	M	Dissection	Ascending to infrarenal	Cecal adenocarcinoma	*In situ*	Died 5 hours postoperatively	
Montoya [26]	1997	78	M	Aneurysm	Descending thoracic	Cecal adenocarcinoma	None	Died 16 hours after admission	
Cohen [27]	1998	77	M	Abscess/dissection	Aortic root/ascending	Cecal adenocarcinoma	Right hemicolectomy	Died 23 days postoperatively	
Johnson [28]	1999	78	M	Aneurysm	Infrarenal	Unknown	None	Died on hospital day 6	
Foga [29]	2000	56	M	Aneurysm	Infrarenal	Crohn's disease	Surgery performed for the aortic aneurysm, but surgical style was unclear	Alive, duration unknown	Unknown
Morrison [30]	2001	71	M	Aneurysm	Thoracoabdominal	Ascending colon adenocarcinoma	*In situ*	Alive at 36 months	Broad-spectrum intravenous antibiotics → penicillin, 8 weeks → oral penicillin, duration unknown
Al Bahrani [31]	2001	63	M	Aneurysm	Infrarenal	Ascending colon adenocarcinoma	Unspecified reconstruction, right hemicolectomy	Alive, duration unknown	Unknown
Munshi [32]	2002	78	M	Aneurysm	Infrarenal	Cecal adenoma	None	Died 1 month later of renal failure	
Zenati [33]	2002	87	M	Aneurysm/dissection	Left subclavian artery to abdominal	Cecal adenocarcinoma	None	Died on hospital day 6	
Liechti [34]	2003	55	M	Aneurysm	Infrarenal	Transverse colon adenocarcinoma	Transverse colectomy, exploration of aorta	Died 5 months after admission	
							Without resection 3 months later		
Takano [35]	2003	69	M	Aneurysm	Infrarenal	Ascending colon adenocarcinoma	*In situ* with omental flap revised to a rectus abdominus flap	Alive at 60 months	Imipenem/cilastatin, duration unknown
							Right hemicolectomy		
Davies [36]	2003	63	M	Aneurysm	Infrarenal	Unknown	Axillobifemoral bypass	Died 2 days postoperatively	
Rucker [37]	2004	77	F	Aneurysm	Infrarenal	Cecal adenocarcinoma	Axillobifemoral bypass, right colectomy	Died 42 days postoperatively	Unknown
Rucker [37]	2004	91	F	Aortitis	Superior mesenteric artery to iliac arteries	Ascending colon adenocarcinoma	Right colectomy	Unknown	
Evans [38]	2004	91	F	Aortitis	Abdominal	Transverse colon adenocarcinoma	Extended right hemicolectomy	Dead at 5 months	
Mohamed [39]	2006	82	M	Aneurysm	Juxtarenal	Ascending colon adenocarcinoma	*In situ* graft, right hemicolectomy	Alive at 15 months	Piperacillin, 8 weeks
Asciutto [40]	2007	71	M	Aneurysm	Juxtarenal	Ascending colon adenocarcinoma	*In situ* graft, right hemicolectomy	Alive at 2 months	Meropenem, 5 days → penicillin G, 25 days, for 30 days total
Laudito [41]	2008	74	F	Aortitis/dissection	Arch	Descending colon adenocarcinoma	Axillobifemoral bypass	Alive, duration unknown	Unknown
Seder [6]	2009	75	M	Aneurysm	Infrarenal	Ascending colon adenocarcinoma	Axillobifemoral bypass, right hemicolectomy	Died 4 months later of recurrent aortitis	56 days total (ciprofloxacin, 14 days; metronidazole, 42 days; vancomycin, 42 days)
Seder [6]	2009	76	F	Aneurysm	Juxtarenal	Cecal adenocarcinoma	Axillobifemoral bypass, right hemicolectomy	Died on hospital day 94	
Gai [42]	2009	76	F	Aortitis/dissection	Arch	Colon adenocarcinoma	Axillobifemoral bypass, hemicolectomy	Unknown	Unknown
Yang [43]	2009	22	M	Aortitis/dissection	Thoracoabdominal	None	None	Died 8 hours after admission	
Moseley [44]	2010	82	M	Aortitis	Infrarenal aorta to right common iliac artery	Cecal adenocarcinoma	Right hemicolectomy	Died 75 days after admission	
Granier [7]	2011	83	M	Aortitis	Descending thoracic	Unknown	None	Died a few hours after admission	
Mao [45]	2011	73	M	Aortitis	Unknown	Ascending colon adenocarcinoma	None	Died	
Demidovich [46]	2012	69	F	Aneurysm	Descending thoracic	Unknown	None	Died a few hours after admission	
Annapureddy [47]	2012	69	M	Aortitis	Infrarenal	Diverticulosis	Axillobifemoral bypass	Alive at 6 months	Oral amoxicillin was administered for chronic suppression therapy
Ge [48]	2012	87	M	Aortitis	Abdominal	Sigmoid adenocarcinoma	None	Dead at 5 weeks	
Khalid [49]	2012	77	M	Aneurysm	Abdominal	Colon adenocarcinoma	*In situ* graft	Alive at 4 weeks	Details are unknown, but chronic suppression therapy was administered via oral antibiotics.
Our case	####	58	M	Aneurysm	Thoracoabdominal	Unknown	Bilateral nephrectomy	Died 10 days postoperatively	
Tokmaji [50]	2013	71	M	Aneurysm	Abdominal	Ulcerative colitis (cancer not investigated)	Right axillofemoral and femoral-femoral bypass	Alive at 2 months, at which time an aneurysm reoccurred	Details and duration are unknown, but intravenous antibiotics were used.
								at the aortic arch; therefore, surgery was performed	
Eplinius [51]	2014	32	M	Aortitis/dissection	Ascending to descending thoracic	None	None	Died	
Al Hadi [52]	2014	63	M	Aneurysm	Arch	Ascending colon adenocarcinoma	Right hemicolectomy	Died 3 days after right hemicolectomy	
Hashimoto [53]	2014	81	M	Aneurysm	Arch/descending	Cecal adenocarcinoma	None	Died 6 days after admission	
Lintin [54]	2014	78	F	Aneurysm	Arch	Ascending colon adenocarcinoma	Endovascular aortic repair and extra-anatomical bypass of the supra-aortic	Alive at 44 months	Details and duration are unknown, but antibiotics were used.
							vessels with a right-to-left common carotid crossover bypass and		
							a left common carotid to left subclavian bypass		
							Right hemicolectomy		
Shah [55]	2015	73	F	Aortitis	Thoracoabdominal	Cecal adenocarcinoma	None	Died 13 days later	
Tabasum [56]	2015	79	F	Aortitis	Ascending/arch	Cecal adenocarcinoma	Endovascular aortic repair right carotid–carotid and	Alive at 61 months	11 days each of meropenem and metronidazole → oral co-amoxiclav duration unknown
							left carotid subclavian bypass		

*F* female, *M* male

#### Sites of aortic aneurysm formation

Aneurysms formed in the ascending aorta/arch in six cases [17, 18, 25, 27, 51, 56], only the arch in four cases [41, 42, 52, 54], the arch/descending aorta in two cases [19, 53], the descending thoracic aorta in four cases [7, 23, 26, 46], and the abdominal aorta in five cases [37, 38, 48–50]. The aneurysms also involved the thoracoabdominal aorta in six cases [24, 30, 33, 43, our case, 55], the infrarenal aorta in 13 cases [6, 16, 20, 28, 29, 31, 32, 34–37, 44, 47], the juxtarenal aorta in four cases [6, 21, 39, 40], both the descending thoracic aorta and the juxtarenal aorta in one case [22], and an unknown location in one case [45].

#### Operative procedures

Of the 24 cases in which surgery was performed, *in situ* grafts were performed in 9 cases [19, 23–25, 30, 35, 39, 40, 49], axillobifemoral bypass in nine cases [6 (two cases), 16, 20, 36, 37, 41, 42, 47], both in si﻿tu grafts and axillobifemo﻿ral by﻿pass in one case [22]﻿, ﻿﻿right axillofemoral and femoral-femoral bypass in one case [50], EVAR in two cases [54, 56], and unknown types of procedure in two cases [29, 31].

#### Prognosis according to operative procedure

The 6-month mortality was 66.7% in cases in which axillobifemoral bypass was performed and 44.4% in cases in which *in situ* graft was performed.

#### Antimicrobial agents

After surgery, antimicrobial agents were administered for 20 of the cases [6, 16, 19, 20, 22–24, 29–31, 37, 39–42, 47, 49, 50, 54, 56]; penicillin was administered in eight of these cases. However, the duration and dosage of antimicrobial agents were unknown [16, 19, 20, 22, 23, 29, 31, 37, 40, 41, 42, 47, 49, 50, 54, 56].

Based on the collective evidence from our case and the published literature, we found that the associated 6-month mortality was extremely high, periaortic gas was present in almost all of the cases, and no standard operative procedure had been established. It was also found that no algorithm for administering antimicrobial agents had been established, and *C. septicum* is associated with cancer in 82.5% of the cases. To improve mortality rates, we propose what are currently considered optimal treatments.

### Early diagnosis in the emergency department

We found that the presence of periaortic gas is frequently concomitant with *C. septicum*-infected aortic aneurysms; thus, the presence of periaortic gas is probably very useful for early diagnosis. *C. septicum*-infected aortic aneurysm progresses quickly, with the aneurysm often expanding within days or weeks; therefore, longer delays before diagnosis result in higher mortality rates [6, 7, 35, 52–54, 56].

However, infected aortic aneurysms are generally rare, accounting for only 0.5 to 1.3% of all aneurysms [57–60]. Infected aortic aneurysms do not present with characteristic findings, making them difficult to diagnose. In addition, the causative agent is chiefly *Staphylococcus aureus* or *Salmonella*; infection by *C. septicum* is even rarer. However, as shown in Fig. 1, *C. septicum*-infected aortic aneurysms also present with periaortic gas in a high proportion (92.6%) of cases, allowing for their early diagnosis. Even in cases without an aortic aneurysm, periaortic gas is sometimes observed, indicating its potential to enable an even earlier diagnosis [37, 38, 44, 47, 48]. Thus, the presence of periaortic gas on a CT scan is a very important sign in this regard.

### Association with cancer

In the cases we reviewed, *C. septicum*-infected aortic aneurysm was associated with cancer in 82.5% of cases, which is higher than the 75% of cases (35% colorectal and 40% hematological) in which *C. septicum* infection had been associated with malignancy, as observed from the results of an earlier report [4]. *C. septicum* proliferates in tissues with a low pH, elevated lactate levels, and low oxygen. These conditions also support the development of ulcerative lesions of the gastrointestinal tract, especially colon cancer, or colitis due to leukemia, neutropenia, and chemotherapy. Infected aortic aneurysms are believed to occur when *C. septicum* enters the bloodstream and triggers a distant infection after adhering to the aortic wall, leading to the assumption of an association with gastrointestinal cancer [5, 23]. Therefore, it is necessary to conduct investigations for gastrointestinal cancer in addition to eradicating the source of *C. septicum*.

### Prognosis according to operative procedure

We found that a standard operative procedure has not been established for the management of *C. septicum*-infected aortic aneurysms. If surgery is performed to treat only the infected aortic aneurysm, the gastrointestinal cancer (the source of *C. septicum*) remains, increasing the risk of reinfection and infection in other sites. Therefore, surgery should be simultaneously performed to treat both the infected aortic aneurysm and cancer, if any. In patients with an unstable condition who cannot undergo simultaneous surgery, failure to perform surgery for the infected aortic aneurysm results in a 6-month mortality rate of 100%; therefore, the infected aortic aneurysm should be treated first.

First, debridement of the focus of the infection (sufficient removal and cleansing of the aneurysmal wall) should be performed. The next step is revascularization; in our review, the 6-month prognosis was 55.5% for an *in situ* graft versus 28.6% for an axillobifemoral bypass, indicating that an *in situ* graft is the superior option. In general, extra-anatomic revascularization of infected aortic aneurysms, such as via an axillobifemoral bypass, has the advantage of not bringing foreign material into the focus of the infection. However, complications frequently occur, including aortic stump blowout (20%), lower limb amputation due to bypass failure (thromboembolism; 20 to 29%), and reinfection (20%) [61]. In addition, mortality rates following operations for infected aortic aneurysm with anatomic revascularization and extra-anatomic revascularization are reportedly 33% and 40%, respectively; thus, anatomic reconstruction yields better outcomes [59]. Many reports have also demonstrated favorable results with *in situ* anatomic revascularization [61–64].

With regards to graft selection, homografts are reportedly superior to prosthetic grafts in inhibiting infection [65, 66]. Also, occlusion and deliquescence occurred in 17% of the cases during the mean 3-year follow-up period following homograft transplantation in a previous study; reoperation for both were reported to be simple, regardless of the infection [65]. The use of rifampin-soaked prosthetic grafts at the infection site has also been reported [67, 68].

Next, we discuss grafts with omental coverage. The omentum, a layer of tissue rich in intraperitoneal vessels and adipose tissue, processes foreign bodies at its milky spots (clusters of monocytes and lymph nodes in the vascular wall); this action is considered to be effective for containing infections. Omental coverage of the area surrounding an *in situ* prosthetic graft and homograft transplantation can prevent the recurrence of infection [63, 69].

For high-risk surgical patients, EVAR is also a viable option [70, 71]. A retrospective European multicenter study found that the 6-month survival rate was 76 to 86% in the case of EVAR for an infected aortic aneurysm; this was lower for non-*Salmonella* infections [72]. Favorable outcomes were obtained via surgery for *C. septicum*-infected aortic aneurysms that involved a combination of EVAR and extra-anatomical bypass of the supra-aortic vessels with a right-to-left common carotid crossover bypass and a left common carotid to left subclavian bypass, followed by surgery for colorectal cancer [54]. A study on surgery for *C. septicum*-infected aortic aneurysm, that involved a combination of EVAR and right carotid–carotid and left carotid-subclavian bypass, followed by surgery for colorectal cancer, yielded a favorable outcome [56]. Thus, a combination of EVAR and surgery for gastrointestinal cancer might improve the prognosis. The outcome in the present case might have been favorable if EVAR was performed.

### Antimicrobial agents

According to the literature, an antimicrobial agent treatment strategy has not yet been established.

#### After surgery for an infected aortic aneurysm

Postoperatively, antimicrobial treatment must be continued. If the aneurysmal wall culture results are known, the range of antimicrobial agents should be narrowed based on the sensitivity patterns of the culture results. The intravenous administration of antimicrobial agents needs to be continued for 6 to 8 weeks following surgery to eliminate an inflammatory response. However, the appropriate duration of subsequent antimicrobial treatment has not yet been established. Although one report stated that postoperative antimicrobial agents administered intravenously could safely be discontinued when the inflammatory response completely subsides [73], the general consensus is that the oral administration of antimicrobial agents should be continued for the rest of the patient’s life (chronic suppression therapy) [58, 74]. In a previous report, despite 12 weeks of antimicrobial agent administration following surgery for a *C. septicum*-infected aortic aneurysm, infection recurred 5 weeks later [24]. Therefore, we support the guideline that the oral administration of antimicrobial agents needs to be continued for the rest of the patient’s life.

#### Prior to surgery for an infected aortic aneurysm

Some authors suggest that surgery should be performed after the infection has subsided [60]. Among the 24 cases of *C. septicum*-infected aortic aneurysm in our review, in which surgery was performed, there were nine cases in which elective surgery and sufficient preoperative administration of antimicrobial agents were performed [6, 20, 22, 35, 36, 39, 47, 54, 56]. The 6-month prognosis in these cases was relatively favorable, at 66.7%. It might be advisable to administer sufficient quantities of antimicrobial agents before performing surgery (at least until the negative conversion of the blood culture is confirmed). This may be done in cases where emergency surgery is unnecessary, such as cases in which infected aortic aneurysm is suspected, but aneurysm formation is either absent or small (diameter < 5.5 cm) or in cases without pain or other symptoms potentially attributable to an aneurysm. The long-term administration of antimicrobial agents can greatly extend survival periods [35, 54]. Of course, as previously stated, aneurysms form rapidly and require careful monitoring and constant preparedness to perform surgery at any time.

### Steps to take when C. septicum is isolated in the blood culture

If there is no contamination, a detailed examination is necessary to determine the presence of an infected aortic aneurysm as well as the presence of gastrointestinal cancer. Even when an infected aortic aneurysm is not detected, careful monitoring for the possible occurrence of an infected aortic aneurysm is necessary if there is no other identifiable cause of the bacteremia.

### Limitations

There are a number of limitations to this study. First, since case reports often involve patients who survive, and we excluded academic conference abstracts, this might have been a source of bias. Therefore, the mortality rate for *C. septicum*-infected aortic aneurysm may have been underestimated. Second, due to the small number of cases, we were unable to assess the long-term prognosis. Long-term prognosis was reported for two patients [35, 54], both of whom underwent elective surgery and received antimicrobial agents for 25 and 41 days prior to surgery. Both cases underwent cancer excision: one case had omental graft coverage [35], and the other case underwent EVAR [54]. These patients survived for 60 months and 44 months, respectively. Given the frequency of comorbid malignant gastrointestinal tumors, long-term prognosis remains an issue for future studies.

## Conclusions

*C. septicum*-infected aortic aneurysm is extremely difficult to treat. However, in this review, we advocate for the need to make an early diagnosis through the identification of periaortic gas, as well as effective treatment options. We believe these could reduce mortality rates.

## Abbreviations

CT: Computed tomography
EVAR: Endovascular aortic repair
